# Characterization of the tumor marker muc16 (ca125) expressed by murine ovarian tumor cell lines and identification of a panel of cross-reactive monoclonal antibodies

**DOI:** 10.1186/1757-2215-2-8

**Published:** 2009-06-18

**Authors:** Cara AR Goodell, Jennifer A Belisle, Jennifer AA Gubbels, Martine Migneault, Claudine Rancourt, Joseph Connor, Muthusamy Kunnimalaiyaan, Rachel Kravitz, Ward Tucker, Michael Zwick, Manish S Patankar

**Affiliations:** 1 Department of Obstetrics and Gynecology, University of Wisconsin-Madison, Madison, Wisconsin-53792, USA; 2 Department of Microbiology and Infectiology, Universite de Sherbrooke, Sherbrooke, Canada; 3 Department of Surgery, University of Wisconsin-Madison, Wisconsin-53792, USA; 4 NeoClone Biotechnology, Madison, Wisconsin-53713, USA; 5 AndroBioSys Inc, 73 High Street, Buffalo, New York 14203-1149, USA

## Abstract

**Objectives:**

The ovarian tumor marker CA125 is expressed on human MUC16, a cell surface bound mucin that is also shed by proteolytic cleavage. Human MUC16 is overexpressed by ovarian cancer cells. MUC16 facilitates the binding of ovarian tumor cells to mesothelial cells lining the peritoneal cavity. Additionally, MUC16 also is a potent inhibitor of natural killer cell mediated anti-tumor cytotoxic responses. Extensive studies using human as well as murine ovarian tumor cell models are required to clearly define the function of MUC16 in the progression of ovarian tumors. The major objective of this study was to determine if the murine ovarian tumor cells, MOVCAR, express Muc16 and to characterize antibodies that recognize this mucin.

**Methods:**

RT-PCR analysis was used for detecting the Muc16 message and size exclusion column chromatography for isolating Muc16 produced by MOVCAR cells. Soluble and cell-associated murine Muc16 were analyzed, respectively, by Western blotting and flow cytometry assays using a new panel of antibodies. The presence of N-linked oligosaccharides on murine Muc16 was determined by ConA chromatography.

**Results:**

We demonstrate that murine Muc16 is expressed by mouse ovarian cancer cells as an ~250 kDa glycoprotein that carries both O-linked and N-linked oligosaccharides. In contrast to human MUC16, the murine ortholog is primarily released from the cells and cannot be detected on the cell surface. Since the released murine Muc16 is not detected by conventional anti-CA125 assays, we have for the first time identified a panel of anti-human MUC16 antibodies that also recognizes the murine counterpart.

**Conclusion:**

The antibodies identified in this study can be used in future purification of murine Muc16 and exhaustive study of its properties. Furthermore, the initial identification and characterization of murine Muc16 is a vital preliminary step in the development of effective murine models of human ovarian cancer. These models will aid in the further elucidation of the role that human MUC16 plays in the etiology and progression of ovarian tumors.

## Background

Epithelial ovarian cancer (EOC) is the fifth leading cause of all female cancer-related deaths in the western world [1]. Despite its prevalence, this disease is marked by difficulties in early diagnosis as well as lack of an effective screening test. The major marker of human EOC is the CA125 peptide epitope, serum levels of which are elevated in EOC patients [2]. The CA125 epitope is contained in MUC16, a 2–5 million Da transmembrane mucin that is over expressed in EOC [3,4]. As a shed type of mucin, MUC16 is both expressed on the cell surface and released following proteolytic cleavage into the extracellular space [5].

Recent studies indicate that MUC16 is not only important as a tumor marker but also promotes peritoneal metastasis of ovarian cancer and suppresses the cytolytic responses of human natural killer cells [6,7]. The physiological function of this mucin is not known; however, its biochemical properties have constrained studies on this molecule. The high molecular weight of MUC16 requires the use of extensive molecular biological approaches to study the importance of this mucin in the pathogenesis of ovarian cancer. In addition, a thorough study of MUC16 expressed in mouse models for ovarian cancer will also aid in understanding its physiological roles.

Recently, several murine ovarian tumor models have been developed [8-10]. In one particular model, transgenic mice were generated expressing the SV40 T-antigen under the direct influence of the Mullerian inhibitory substance (an ovary-specific gene), and the mice spontaneously developed ovarian cancers resembling poorly differentiated ovarian adenocarcinomas in women [8,11]. Murine ovarian tumor cell lines, designated as MOVCAR, have been generated from these tumors [8]. These cell lines provided us an opportunity to perform biochemical and physiological studies on the murine counterpart of MUC16, designated as Muc16. Here we report the expression and initial biochemical characterization of Muc16 expressed by the MOVCAR cells. Specifically, we identify expression of Muc16 mRNA and provide evidence that, unlike MUC16, the murine ortholog is not expressed on the cell surface but is instead primarily released from the MOVCAR cells. In addition, we have for the first time identified specific monoclonal antibodies that can be used in future studies of murine Muc16.

## Methods

### Cells, antibodies, and other reagents

The anti-MUC16 antibody VK8 [12] was a kind gift from Beatrice Yin (Memorial Sloan Kettering, New York, USA). The panel of anti-MUC16 mouse monoclonal antibodies was generated against human ascites derived MUC16 using the ABL-MYC transformation technology [13,14]. The four murine ovarian cancer cell lines–MOVCAR 1, 2, 9, and 10–were kindly provided by Dr. Denise Connolly (Fox Chase Cancer Center, Philadelphia) and cultured in DMEM supplemented with 10% FBS, 0.2% ITS and 1% antibiotic-antimycotic. The human epithelial ovarian tumor cell lines OVCAR-3, SKOV-3, and CAOV-3 were purchased from ATCC.

### RT-PCR

Total RNA was isolated from MOVCAR cell lines using the Qiagen RNeasy^® ^Mini kit and 2 μg of total RNA was reverse transcribed. PCR reactions were performed with 2.5 μL of cDNA. For each sample, a control tube containing all reagents except template cDNA was prepared. cDNA was amplified with the following primer pairs from Integrated DNA Technologies: Muc16 5'-TGCCACCTACCAGTTGAAAG-3' and 5'-GTACCGCCAAGCAGATGAG-3'; GAPDH 5'-TGCTGAGTATGTCGTGGAGTCTA-3' and 5'-AGTGGGAGTTGCTGTTGAAGTCG-3'. The amplified Muc16 cDNA from MOVCAR-2 cells was sequenced at the University of Wisconsin-Madison Biotechnology Center.

### Flow cytometry

Cells (2.5 × 10^5^) were fixed with 2% paraformaldehyde, washed three times with sterile filtered PBS/1% BSA (PBS-BSA), and permeabilized with 0.1% Triton X-100 on ice. Unfixed cells (2.5 × 10^5^) were kept on ice during this time. All cells were incubated with primary and secondary antibodies for 30 minutes on ice. The 618F and 653F antibodies were used at 1:250 dilutions in PBS-BSA. VK8 from cell culture supernatant was used directly for labeling. The FITC-conjugated goat anti-mouse (GAM) IgG, Fc specific secondary antibody (Jackson ImmunoResearch) was used for detection at 1:100.

### Protein isolation, electrophoresis, and Western blotting

Soluble Muc16 was isolated from MOVCAR-2 serum-free spent media which was concentrated 20-fold. Approximately 5 mL of concentrated media was loaded onto a Sepharose-CL4B (Sigma) column (1.5 cm × 42 cm) pre-equilibrated with 10 mM ammonium bicarbonate buffer and 1 mL collected fractions were monitored for absorbance at 280 nm. The desired fractions were pooled and lyophilized. Soluble human MUC16 was isolated as described in our previous study [15]. Cell lysates were prepared by treating the ovarian tumor cells with Tris buffered saline containing 0.5% Triton X-100 and a cocktail of protease inhibitors (Sigma).

For Western blotting, 25 μg of protein was separated on 7.5% SDS-PAGE gels and electroblotted on a PVDF membrane. The membranes were sequentially overlaid with anti-human MUC16 antibodies followed by horseradish peroxidase labeled GAM IgG (Pierce; 1:20,000 dilution). Signals were detected by using the West Pico kit (Pierce). Coomassie Blue and silver staining of gels was performed using protocols established in our laboratory.

### ConA Chromatography

Concentrated harvest media from MOVCAR-2 cells containing 22 mg of total protein was loaded onto a 5 mL Concanavalin A (ConA) column (Sigma; ConA immobilized on 4% cross-linked agarose) equilibrated in 1× ConA buffer (100 mM Tris HCl containing 1.5 M sodium chloride, 10 mM calcium chloride, 10 mM magnesium chloride, and 0.2% sodium azide). The column was eluted with ConA buffer and fractions were monitored for absorbance at 280 nm. The bound glycoproteins were eluted by washing with a step gradient (100, 250, and 500 mM) of α-methylmannopyranoside (α-Me-Man). Fractions from eluted peaks were pooled, and the proteins were analyzed by Western blotting.

## Results

### Expression of Muc16 mRNA in MOVCAR cells

**Figure 1:**
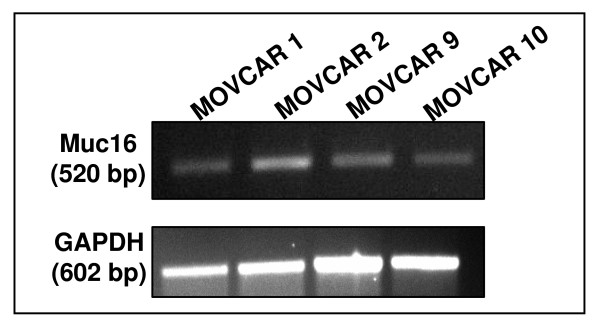
**RT-PCR verification of Muc16 mRNA expression in four MOVCAR cell lines**. GAPDH gene was used as a housekeeping control.

We first conducted RT-PCR experiments to determine expression of Muc16 by the MOVCAR cells. Specific primers for Muc16 were designed around the unique domain which was identified based on its percent identity with the corresponding region of MUC16. The region between 25605 bp to 26125 bp of the Muc16 sequence reported in GenBank accession no. XM_911929.2 was amplified by these primers. Muc16 mRNA was expressed in each of the four MOVCAR cell lines tested by RT-PCR (Fig. 1). After DNA sequencing, the PCR product from the MOVCAR-2 cell line was found to have 99% identity with the projected sequence of Muc16. Although Muc16 was always expressed in these cells, the level of Muc16 mRNA varied in different passages (data not shown).

### Detection of Soluble Muc16 in MOVCAR media

Soluble MUC16 is shed into the spent harvest media of the human epithelial ovarian tumor cell line, OVCAR-3. The shed MUC16 can be isolated from the media following concentration and separation by size exclusion chromatography [15]. We therefore determined if Muc16 was present in the spent media from MOVCAR-2 cells.

**Figure 2:**
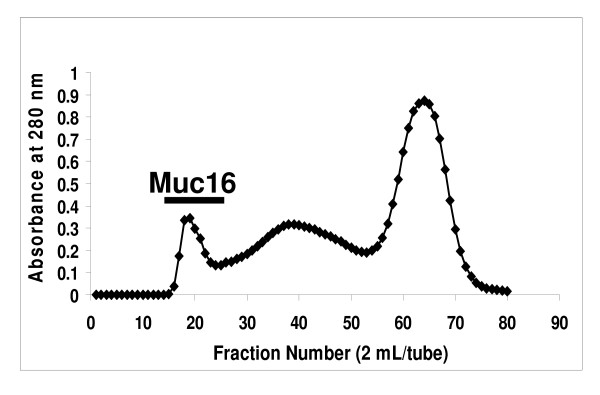
**Purification of Soluble Muc16 on a Sepharose CL-4B Column**. Concentrated MOVCAR-2 spent media was separated as described in Methods. Fractions located under the bar stained positive for mucin with Alcian Blue and Western blotting.

We consistently found that the MOVCAR-2 media purification profiles on a Sepharose CL-4B size exclusion column followed the same pattern as the OVCAR-3 media profiles (Fig. 2) [15]. The murine Muc16 was only slightly retarded on this column (Fig. 2) and was initially identified (data not shown) by Alcian Blue staining [16].

**Figure 3:**
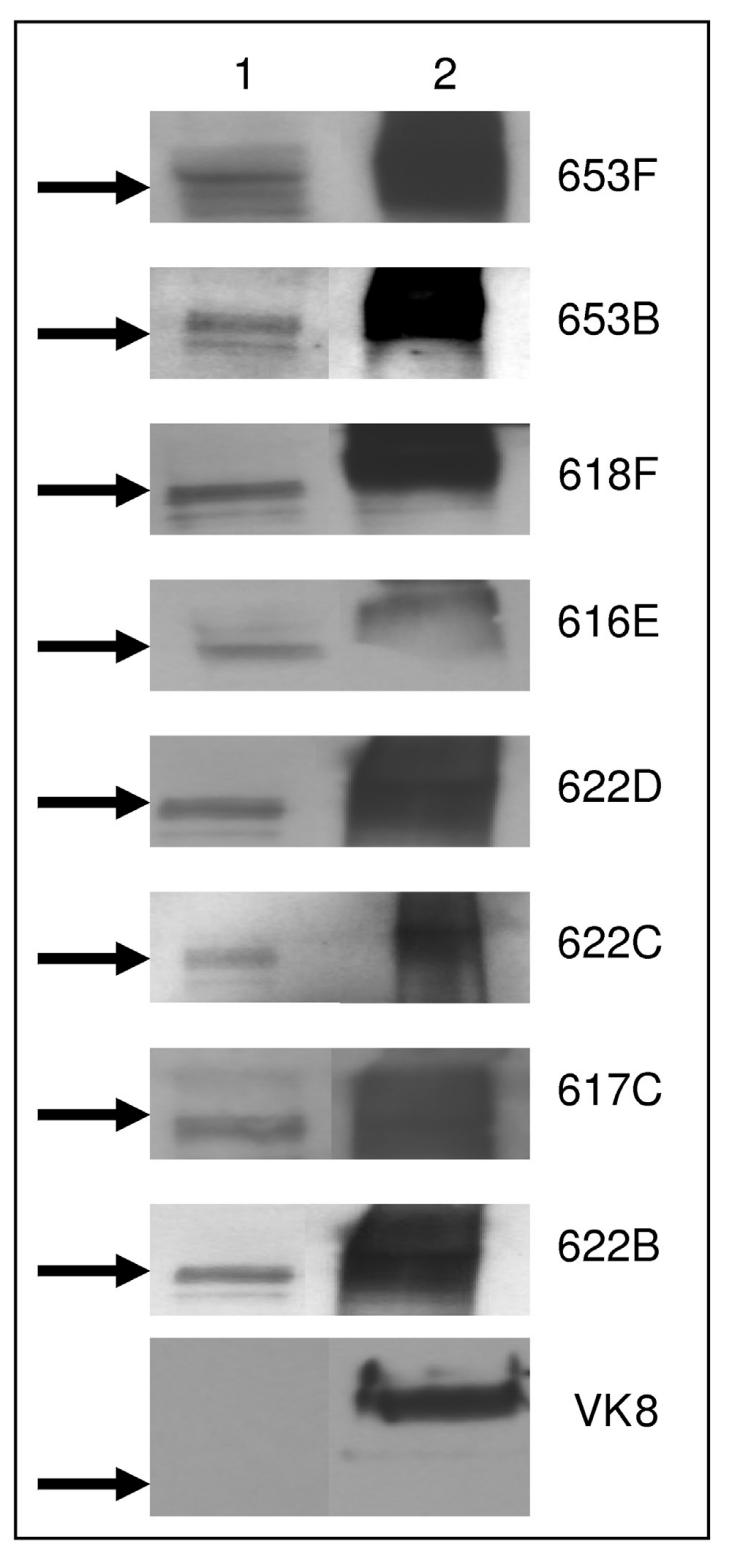
**Identification of soluble Muc16 and MUC16 by Western blotting**. Purified MUC16 (25 μg total protein/lane) from MOVCAR-2 (lane 1) and OVCAR-3 cells (lane 2) was electrophoresed by SDS-PAGE and probed with a panel of anti-MUC16 monoclonal antibodies. Arrows indicate migration of 250 kDa molecular weight marker and identity of antibody used is shown on the right of each blot.

To specifically identify Muc16, we conducted Western blot analysis of Pool 1 using MUC16 specific VK8 and OC125 antibodies. No bands for Muc16 were detected in these analyses. The widely employed clinical serum CA125 assay was also unable to detect CA125 in spent media of MOVCAR-2 cells. We therefore tested a panel of ten anti-MUC16 antibodies that we recently generated using the novel ABL-MYC technology. All of these antibodies were able to detect an approximately 250 kDa band for Muc16 (Fig. 3). The binding to Muc16 was weaker as compared to MUC16 and was usually observed at 1:250 dilution of the primary antibodies.

### 618F and 653F specifically recognize human MUC16

**Figure 4:**
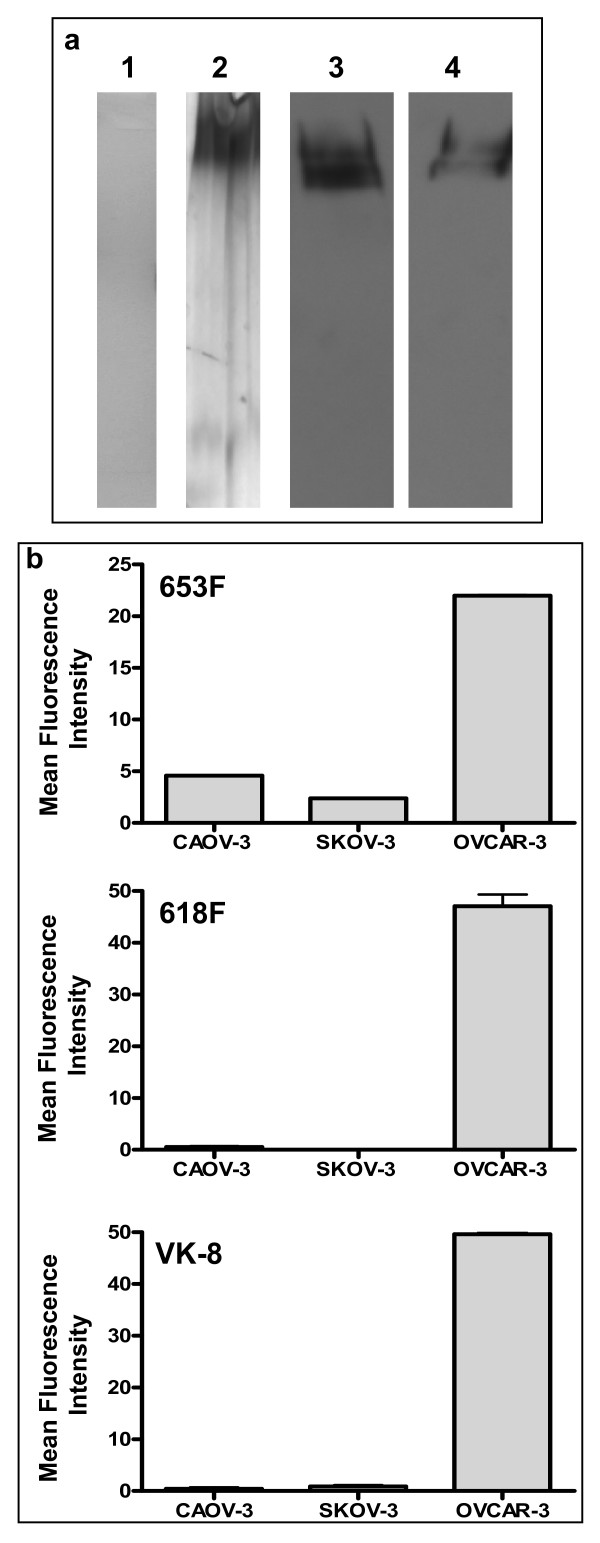
**Specificity of 618F for human MUC16**. (A) Purity of human MUC16 isolated from spent media of OVCAR-3 cells as determined by Coomassie Blue (1) and silver staining (2) of SDS-PAGE gel. Western blot analysis of the purified human MUC16 was conducted using the 618F (3) and the VK-8 (4) antibodies. (B) MUC16 expression on OVCAR-3, CAOV-3, and SKOV-3 was determined by flow cytometry using 653F, 618F, or VK-8 as the primary antibodies. Mean fluorescence intensity of the corresponding isotype controls was subtracted in each case. Data shown is mean of two independent experiments. Note that the binding of 618F, 653F and VK-8 to these three cell lines is comparable.

Two antibodies from the panel that efficiently recognized Muc16 by Western blotting were 618F and 653F. When a purified preparation of human MUC16 was analyzed by Western blotting, the 618F antibody exhibited a similar banding pattern to that shown by the VK-8 antibody (Fig. 4A). Using flow cytometry we also demonstrated that both 618F and 653F specifically bind to the OVCAR-3 but not to the MUC16^neg ^SKOV-3 or CAOV-3 cells (Fig. 4B). The binding of 618F, 653F and VK-8 to the OVCAR-3, SKOV-3, and CAOV-3 cells was comparable. Considering the demonstrable specificity of 618F and 653F for MUC16 and their ability to recognize Muc16 from the MOVCAR-2 spent media, we primarily conducted all of our further experiments with these two antibodies.

### Binding of murine muc16 to the Lectin ConA

**Figure 5:**
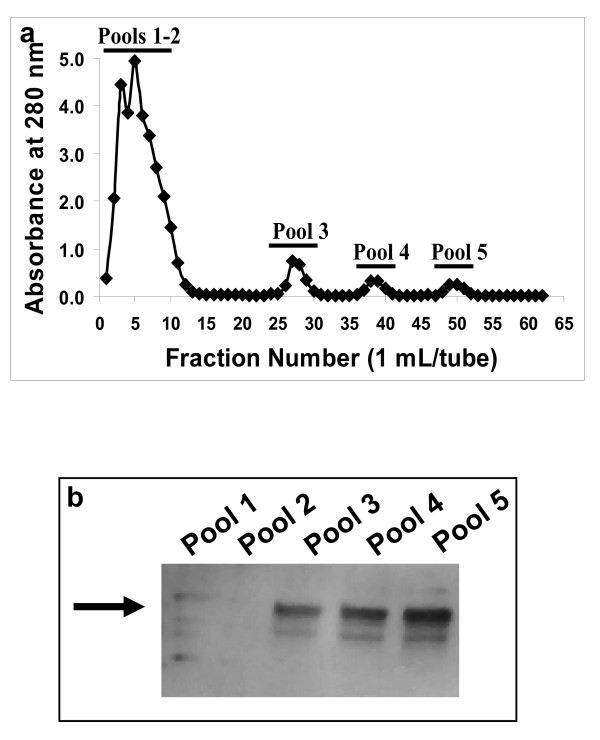
**Binding of murine Muc16 to the lectin ConA**. (A) Concentrated MOVCAR-2 spent media was separated on a ConA affinity column. Fractions eluted with ConA buffer were combined in pools 1 and 2. Fractions eluted with 100, 250, and 500 mM α-Me-Man concentrations were combined in pools 3–5, respectively. (B) Pooled fractions were electrophoresed by SDS-PAGE and probed with 653F. The arrow indicates migration of 250 kDa molecular weight marker. Murine Muc16 was detected primarily in pools 3–5.

Although mucins are known to express high amounts of O-glycans, MUC16 also carries a significant proportion of N-linked oligosaccharides. To determine if Muc16 also expresses N-linked glycans, we loaded the concentrated spent media from MOVCAR-2 cells on a ConA column. The flow-through fractions from the column were collected and the retained material was eluted using sequential washes containing increasing concentrations of α-Me-Man (Fig. 5a). Muc16 was detected in all of the α-Me-Man fractions but not in the flow-through pool by Western blot analysis using the 653F antibody (Fig. 5b).

### Murine muc16 is not expressed on the MOVCAR cell surface

**Figure 6:**
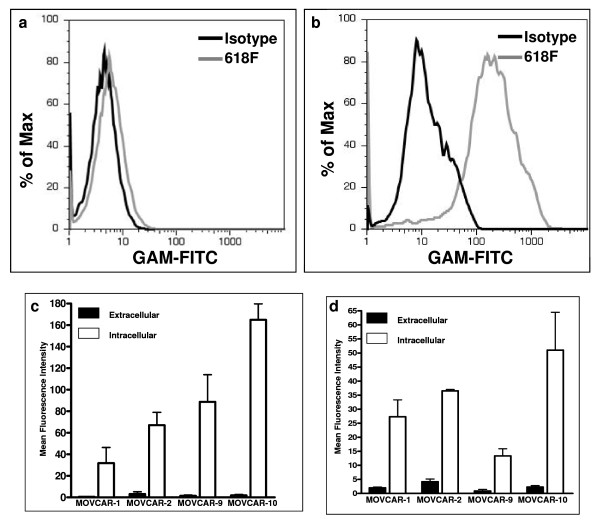
**Extra-and intracellular Muc16 expression by MOVCAR cells**. (A) MOVCAR-10 cells were labeled with 618F (grey line) and analyzed for cell surface expression of Muc16 by flow cytometry. Isotype control is shown by dark line. (B) MOVCAR-10 cells were fixed and expression of intracellular Muc16 was detected by using the 618F antibody (grey line). Dark line shows isotype control. (C) Expression of cell surface and intracellular Muc16 in the four MOVCAR cell lines was determined by flow cytometry using the 618 F antibody. The mean fluorescence intensity for the binding of 618F to the cell surface and intracellular Muc16 is plotted after subtracting the mean fluorescence intensity for the matched isotype controls. Each measurement is a mean of two independent experiments. (D) Same as in (C) except 653F was used for detection of murine Muc16.

Having identified soluble forms of Muc16 by Western blotting, we investigated whether this mucin was also expressed on the cell surface of MOVCAR cells. We consistently found little to no extracellular Muc16 expression on the MOVCAR-10 cells when the expression of this mucin was determined by flow cytometry using the 618F antibody (Fig. 6a). On the other hand, high levels of intracellular Muc16 were detected in the MOVCAR-10 cells using the 618F antibody (Fig. 6b). These results are in sharp contrast with the intense extracellular binding of this antibody found on OVCAR-3 cells (Fig. 4b).

Correcting for background fluorescence of the isotype control, our results for all MOVCAR cell lines showed a clear expression of intracellular Muc16 and only minimal presence of Muc16 on the cell surface (Figs. 6a–d). Similar results were obtained with both the 618F and 653F antibodies (Fig. 6c and 6d).

## Discussion

We have identified soluble and cell-associated Muc16 in MOVCAR cells. While soluble MUC16 is over 3 million Da, Western blots indicate that the murine counterpart is significantly smaller, at approximately 250 kDa. With its tertiary structure intact during size exclusion chromatography, however, Muc16 behaves as a much larger protein. This suggests intermolecular crosslinking in Muc16 similar to that observed in other mucins. Extensive glycosylation of Muc16 arising from the presence of O-linked and N-linked oligosaccharides, as demonstrated in our studies, may also contribute to its large tertiary structure.

Given the lack of cell surface Muc16 on MOVCAR cells, we can make important distinctions between the human and murine forms of the mucin. MUC16 is both expressed on the cell surface and shed from the cell in soluble forms. Muc16, on the other hand, is detected primarily in the spent media and in the intracellular environment. This observation indicates that the Muc16 is either rapidly cleaved from the cell surface by a very active proteolytic enzyme or is an alternatively spliced form that is primarily secreted by the MOVCAR cells. Our future studies will focus on deciphering the mechanisms that lead to the generation of the shed Muc16.

The shed and cell surface bound MUC16 play important roles in the progression of human ovarian tumors. While the shed MUC16 appears to have major influence on the cytolytic function of natural killer cells, the cell surface bound MUC16 is important for binding of the ovarian tumor cells to the mesothelial cells that line the peritoneal cavity. Since the MOVCAR cells shed Muc16, this murine model may be important in understanding the immunomodulatory roles of this mucin. The shed Muc16 should also be found in the serum of mice bearing the MOVCAR tumors. The antibodies identified in the current study can therefore be used to monitor tumor progression in mice. These antibodies can also be used to purify Muc16 so that its biochemical and biological properties can be exhaustively studied.

## Conclusion

In this study we demonstrate that Muc16 is expressed by murine ovarian tumor cells and can be detected by newly developed murine monoclonal antibodies that were initially generated against human MUC16.

## List of abbreviations

EOC: epithelial ovarian cancer; ConA: Concanavalin A; α-Me-Man: α-methylmannopyranoside.

## Competing interests

Anti-MUC16 antibodies used in this study were developed for commercialization by Neoclone Biotechnology. Dr. Zwick was employed at Neoclone Biotechnology when this study was conducted. The University of Wisconsin-Madison researchers have no competing interests to declare.

## Authors' contributions

CARG conducted the RT-PCR and western blot ananlysis and was assisted in these experiments by JAB and JAAG. MM and CR helped in designing appropriate Muc16 primers. JC assisted in obtaining and maintaining murine ovarian tumor cells. MK assisted in standardizing the RT-PCR protocols. RK, WT, and MZ were involved in the development of the anti-MUC16 antibodies. MSP designed this study and developed the manuscript.
